# Evaluation of vaccine storage and distribution practices in rural healthcare facilities in Kenya

**DOI:** 10.1186/s40545-023-00535-2

**Published:** 2023-02-21

**Authors:** Dennis Kipkoech Sinnei, Peter Ndirangu Karimi, Shital Mahindra Maru, Stephen Karengera, Thomas Bizimana

**Affiliations:** 1 East African Community Regional Centre of Excellence for Vaccines, Immunization and Health Supply Chain Management, College of Medicine and Health Sciences, University of Rwanda, Kigali, Rwanda; 2 Department of Pharmacy, School of Pharmacy, University of Nairobi, Nairobi, Kenya

**Keywords:** Vaccines, Cold chain, Rural health facilities, Distribution, Storage

## Abstract

**Background:**

Vaccines require cold chain storage conditions, and good distribution practices throughout the supply chain to maintain their quality and potency. However, in the last mile of the vaccines supply chain, these requirements may not be guaranteed resulting in reduced effectiveness which could lead to an upsurge in vaccine preventable morbidity and mortality. The aim of this research was to evaluate vaccine storage and distribution practices in the last mile of vaccine supply chain in Turkana County.

**Methods:**

A descriptive cross-sectional study was conducted from January 2022 to February 2022 across seven sub-counties in Turkana County, Kenya, to assess vaccine storage and distribution practices. The study sample size was 128 county health professionals across 4 hospitals, 9 health centers, and 115 dispensaries. The respondents were selected using simple random sampling within the facilities strata. Data were collected using a structured questionnaire, adapted, and adopted from a standardized WHO questionnaire on effective vaccines management and administered to one healthcare personnel working in the immunization supply chain per facility. Data were analyzed using excel and presented as percentages in table forms.

**Results:**

A total of 122 health care workers participated in the study. Most respondents (89%, *n* = 109) had utilized a vaccine forecasting sheet, but only 81% did have an established maximum–minimum level inventory control system. Many of the respondents had sufficient knowledge of ice pack conditioning although 72% had adequate vaccine carriers and ice packs. Only 67% of respondents had a complete set of twice-daily manual temperature records at the facility. Most refrigerators complied with the WHO specifications but only 80% of them had functional fridge-tags. The number of facilities that had a routine maintenance plan was below average while only 65% had an adequate contingency plan.

**Conclusion:**

Rural health facilities have suboptimal supply of vaccine carriers and icepacks for effective storage and distribution of vaccines. In addition, some vaccine fridges lack functional fridge-tags for proper temperature monitoring. Routine maintenance and contingency plans remain a challenge to ensure optimal service delivery.

## Background

According to World Health Organization (WHO), vaccines save lives by preventing 3.5–5 million deaths each year through immunization [1]. Vaccines require special storage conditions called cold chain. Most ought to be stored and distributed at a temperature of between + 2℃ and + 8℃ throughout their supply chain to maintain their quality and potency [2]. According to the 2016 WHO and United Nations Children’s Fund (UNICEF) joint report, only 15% of Low- and Lower-Middle-Income Countries (L&LMIC) achieved the recommended practice for effective vaccine distribution down the supply chain [3].

In Kenya, the cold chain distribution system from the central store to the service delivery point resembles what was observed by Chen et al. [4]. This distribution model has been shown by Lim et al. to be based on previously existing administrative structures rather than cost efficiency [5]. Besides, the complexity of the pipeline for vaccines from the regional depot to the facility level may create breaking points in cold chain maintenance due to inadequate infrastructure, and skills gap [6].

In Nairobi city county Kenya, only 1% of the public health facilities have a reliable designated utility vehicle to collect vaccines and other supplies from the sub-county depots [7]. A similar situation is mirrored in Turkana County where the average distance a person needs to travel to access the nearest health facility for vaccination was found to be 15 km in 2017 [8] against the 5 km recommended by WHO. Moreover, these transportation challenges contribute to vaccines stockout at public health facilities.

Besides, traveling to rural health facilities is difficult [9]. Therefore, transporting vaccines to these facilities from the county depot/sub-county to the vaccination facility is a logistic challenge. Healthcare workers in rural health facilities store and transport vaccines from the county/sub-county depot in vaccine carriers which use icepacks [10].

The disadvantage with these carriers is that they have limited storage capacity [6]. Besides, inadequate conditioning of ice packs before storage and transporting the vaccines can freeze the vaccines [11]. This could lead to wastages or if administered to a client, it could fail to elicit an immune response which may require revaccination [12].

Tremendous improvements in the delivery of vaccines supply and the cold chain system have been made at the national level. This includes outsourcing of transportation services of vaccines from the national to the regional stores using refrigerated truck which has improved the temperature condition during transportation, quantity of vaccines and overall efficiency [13]. However, at the county level, this has not been matched.

Therefore, this research sought to evaluate the storage and distribution practices among healthcare workers coordinating vaccine supply chain in rural healthcare facilities in Turkana County.

## Methodology

### Study location and research design

This study was conducted in Turkana County, Kenya, which is among the northern arid vast counties with difficult geographical terrain in reaching rural health facilities. A descriptive cross-sectional study was carried out across the sub-county strata targeting a population size of one hundred and eighty-nine (189) health care workers, one per facility. Turkana County has seven administrative sub-counties. Immunization services are offered in health facilities and outreach sites linked to health facilities within the seven sub-counties. These facilities include dispensaries, health centers, sub-county hospitals and county hospitals. Immunization services are offered by mostly nurses and Expanded Program of Immunization (EPI) logisticians who are stationed at these facilities.

### Target and study population

Vaccine storage and distribution by health care workers occur within the seven sub-counties. These seven sub-counties formed the strata where the study sample was obtained. In these strata, the facilities were classified according to their level of care, i.e., hospitals, health centers and dispensaries to form 3 sub-strata within each stratum. This classification gave total distribution of 189 immunizing health facilities across the seven strata where the study participants are located. After applying Yamane’s formula from the finite study population of 189 and a margin of error of 5%, the study sample obtained was 128 as shown below:

$$n=\frac{N}{1+N{e}^{2}},$$

where N = study population = number of health facilities where respondents that will be interviewed, n = sample size, e = level of significance = 0.05,

$$n=\frac{189}{1+189{(0.05)}^{2}}$$

$$n=128.$$

A proportional ratio (sample size/total population) *stratum size) was then applied to each of the 3 sub-strata across the seven strata to determine the number of study participants per level of care. This resulted to 4 hospitals, 9 Health centers and 115 dispensaries as the strata sample size. To select the health facilities stratified random sampling was used and one respondent was chosen from each.

### Data collection

Research assistants were trained online via google meet for 2 h on how to fill the closed ended structured questionnaire adapted from the tool developed by WHO for vaccine management assessment [14] in a google form format. The questionnaire had four sections which included vaccine storage and distribution, vaccine management, cold chain equipment infrastructure and management and vaccine availability. Each category had a range of about 5 to 33 questions which were scored against a recommended score of 80% and above by WHO on effective vaccine management.

The questionnaire was then pre-tested in a pilot study conducted in conveniently sampled seven facilities in which one healthcare worker per facility was interviewed. These seven healthcare workers were not included in the final sampled population. Feedback from the team was then analyzed and incorporated in the final questionnaire to make it comprehensive.

During the data collection process from January 7th to February 28th, 2022, interviews tested the knowledge and practices of the healthcare workers and scored against recommended WHO standard practices. Besides, direct observations were made to available records and data were extracted accordingly and scored. From a target of 128, only 95% of the respondents were reached by the research assistants. The 5% that was not reached was because of some respondents were not at the facility during the visit due to being on leave, off, sick, and insecure areas at the time of the study.

### Data management and analysis

The google form structured questionnaires completed by the research assistants were sent directly into the researcher’s email. The data were extracted using a google spreadsheet Excel 2019. It was then cleaned, analyzed for completeness, and stored in the researcher’s google drive and zipped to protect it. The spreadsheet was coded and analyzed in excel for frequency and percentages. Then, the results were presented in the form of tables.

## Results

The vaccine storage and distribution were assessed by asking the participants relevant questions to the participants**.** Most of the health professionals (89%) knew how to condition icepacks as shown in Table 1. Unfortunately, only 72.9% of ice packs and vaccine carriers were qualified to be adequate for vaccine storage and transportation. In addition, it was observed that only 73% of health facilities had vaccine carriers that could accommodate peak vaccine stock level during requisition.

**Table 1:** Storage and transportation of vaccines to rural healthcare facilities

Characteristic	Status	Number (*n* = 122)	Percentage (%)
Adequacy of vaccine carriers and ice packs	Adequate	89	72.95
Knowledge of staff on how to condition ice packs	Know	109	89.34
Availability of vaccine carrier/cold box to accommodate peak vaccine stock level during requisition	Adequate	90	73.77
Mode of transport used to deliver vaccines to the health facility	Car	47	38.52
	Motor bike	65	53.28
	Boat	10	8.20
Deliveries completed on time during the past 6 months	On time	89	72.95

Absolute majority of the immunization nurses had a filled vaccine forecasting sheet but only 58% of them used a counter requisition and issue voucher. The vaccine order fill rate was observed to be at 72.95% with majority documenting the vaccine vial monitor (VVM) status in stock ledgers during receipt of the vaccines at the stores as shown in Table 2. Despite a great number of the immunization nurses having been reported to be knowledgeable on detecting frozen vaccines during transportation, only 67% could vividly describe the shake test.

**Table 2:** Cold chain management practices at the health facilities

Characteristics	Status	Number (*n* = 122)	Percentage (%)
Availability of a utilized standardized forecasting tool for the staff to calculate their vaccine needs	Available	109	89.34
Use of a counter requisition and issue voucher during requisition of the vaccine orders	Used	71	58.20
Vaccines issued as requested	Yes	89	72.95
Temperature recording during receipt of vaccines at the health facility	Recorded	91	74.59
Recording of VVM status during receipt of vaccines at the health facility	Recorded	112	91.80
Knowledge of the facility staff on the ability to detect frozen vaccines during transportation	Able	99	81.15
Ability of the staff to conduct a freeze test	Able	82	67.21
Distribution of vaccines with matching quantity of diluents	Matches	114	93.44
Documentation of vaccine losses in stock records	Being done	54	44.26
	No data available	25	20.49
Vaccine loss of less than 1% due to incorrect transport conditions from the supplying store	Yes	93	76.23
Awareness of the staff on correct storage temperature range of vaccines	Aware	115	95.08
Ability of the staff to give the correct temperature range of frozen vaccines	Able	108	88.52
Availability of twice daily manual temperature records for vaccines	Available	82	67.21
Vaccine arranged in trays as per recommendation	Yes	113	92.62
Updates on vaccine receipts and dispatches	Being done	114	93.44
Documentation of vaccine and diluent vial sizes	Done	103	84.43
Recording of vaccine and diluent batch/lot numbers	Recorded	119	97.54
Recording of vaccine and diluent expiry dates	Recorded	120	98.36
Vaccine distribution done according to FEFO principle	Yes	118	96.72
Ability of staff to make exceptions to FEFO during issuing	Able	116	95.08
Conducting and recording physical counts during the past 6 months	Documented	110	90.16
Vaccines tallying with what is documented on the ledger	Tallies	103	85.12
Vaccine store clean and free from pest infestation	Yes	119	97.54
Vaccines being stored correctly, i.e., no freeze sensitive vaccines stored close to the evaporator plate	Yes	112	91.80
Use of matching diluents with vaccines	Yes	120	98.36
Diluents always kept in cold chain before and during every immunization session	Cooled	115	94.26
Vaccine carriers containing foam pads	Contains	53	43.44
Availability of well displayed IEC material on VVM interpretation	Available	97	79.51
Ability of health worker to read and interpret vaccine vial monitor (VVM)	Able	121	99.18
Ability to use VVM status for vaccine management, i.e., stage II vaccines used first	Able	121	99.18
Adoption of the multi vial dose policy (MDVP) at the facility during sessions	Adopted	99	81.15
Ability of the health worker to explain on how to use the MDVP	Able	94	77.05

In majority of the vaccine stores, vaccines were arranged in trays as per the recommended standards [18] but only 67% of the vaccine stores had a set of twice daily manual temperature records observed. Documentation of both vaccine type, diluent vial size and expiry dates were also being done by majority of the immunization nurses. Documentation of physical counts and distribution of vaccines according to First Expiry First out (FEFO) principle, was done by a greater majority of the healthcare workers who were also able to make exceptions to this principle based on the VVM stage of vaccines. However, at the service delivery point, only 43% of the vaccine carriers had foam pads.

It was noticed that most vaccine stores had functional vaccine refrigerators that complied with WHO specification [19] and with functional fridge-tags as shown in Table 3 below. However, most stores had a mediocre rating in keeping an up-to-date inventory list, routine and preventive maintenance plan, and emergency contingency plans with visible contacts in case of equipment failure. Despite having functional refrigerators, most had over 5 years in service.

**Table 3:** Cold chain infrastructure and adherence to a planned preventive maintenance

Characteristics	Status	Number (*n* = 122)	Percentage (%)
Compliance of vaccine refrigerator with WHO recommendation	Yes	100	81.97
	No refrigerator	4	3.28
Functionality of refrigerators at the time of inspection	Functional	107	87.70
	No refrigerators	4	3.28
Functional fridge-tags stored with vaccines in refrigerators	Functional	98	80.33
	No refrigerators	3	2.46
Vaccine refrigerators maintaining a temperature range of + 2 to + 8	Maintains	99	81.15
	No refrigerator	4	3.28
Vaccine freezers maintains a temperature range of − 15 to − 25	Yes	44	36.07
	No freezer	36	29.51
Availability of voltage regulators on electrical refrigerators	Available	53	43.44
Availability of an equipment inventory list for cold chain equipment at the facility	Available	71	58.20
Availability of a routine maintenance plan or a preventive maintenance schedule for cold chain equipment at the facility	Available	58	47.54
Adequacy of contingency plans in case of an equipment failure	Adequate	83	68.03
Emergency contacts visibly posted on the vaccine store	Yes	57	46.72
Staff capacity to respond to excursions	Adequate	91	74.59
Cold chain equipment failure within the last 6 months	No	74	60.66
Length of time taken to repair the equipment failure	0-7 days	73	59.84
	7–14 days	9	7.38
	14–30 days	8	6.56
	Over 1 month	32	26.23
Length of time refrigerators have been used	0–5 years	46	37.70
	5–10 years	64	90.16
	Over 10 years	12	9.84

Majority of the stores having established and documented the minimum and maximum stock levels in the vaccine stores and had only 39% of the facilities reported to have experienced vaccine stock outs in the past six months as shown in Table 4 below.

**Table 4:** Vaccine availability and management practices at the health facility

Characteristics	Variable	Number (*n* = 122)	Percentage (%)
In the past 6 months, has the facility experienced stockouts?	Experienced stock outs	48	39.34
Check if the facility has established and documented maximum and minimum stock levels in the store	Established	100	81.97
Instances where the safety stocks were consumed for the facility	Yes	39	31.97
Ascertain if the vaccines tallies with the diluents by conducting a random sample	Tallies	108	88.52
Wastages documented in the stock ledger were not more than 1%	Less than 1%	63	51.64

## Discussion

The aim of the study was to evaluate the vaccine management practices to rural health facilities in Turkana County, Kenya against WHO recommended standards on effective vaccine management [20]. Similarly, to what was observed in Nigeria [21], nurses in Kenya know how to condition icepacks but a different situation was observed in rural Ghana at 47% [22]. Despite these differences, the 11% of nurses who do not know how to condition ice packs may use frozen icepacks which may freeze vaccines during storage and distribution as observed by Hanson et al. [23]. To improve the knowledge and practices on vaccine managers, several studies recommend in-service training, mentorship and continuous supportive supervision [21, 22, 24].

Motorbikes were found to be the major means of transport used by immunization nurses to collect vaccines from the sub-county depot to rural health facilities in Turkana county, which corroborates with a previous study done by Bogale et al. in Ethiopia [25]; where it was observed that about half of the facilities sampled were using motorcycle to transport vaccines. But, this cannot be generalized to all health facilities because there are areas that are easily accessible by other means of transport. Lydon et al. [26] identified non-vaccine costs such as human resource, fuel and motorbikes to be among the in-country supply chain costs. The inability to allocate adequate funds for fuel may often lead to delays in deliveries and inevitably to stock outs [27].

Healthcare workers in Kenya were able to utilize the vaccine forecasting sheet, but in contrast only about a half utilized a counter requisition and issue voucher. These gaps could be due to the unavailability of these tools and inadequate knowledge of the users to fully utilize them. To address this gap, integrating technology with the tools and building the capacity of healthcare workers on the utilization of tools could help to improve the reordering process and track the order fill rate. This would influence vaccines availability at the service delivery points as observed in Nigeria [28].

Monitoring of temperature excursions during transportation at the serve delivery points was not optimum in both Kenya, and the same situation was observed in the United Mexican states [29]. This may be due the use of vaccine vial monitors to receive vaccines at end user point, although it is not the best method. A study conducted in Ghana recommended that standard operating procedures for reference should be made available at the facility to improve vaccine management procedures [22], introduction of package level freeze and heat indicators as proposed by Falcon et al., in order to help reduce wastage by discarding vaccines on suspicion of having been exposed to freezing conditions [29].

In terms of documentation of twice-daily manual temperature records, it was observed that tools were not consistent in completing them and this was comparable to findings from the study conducted by Lutukai et al. [30]. Not only understaffing and unavailability of a functional temperature monitoring tool may be possible, but also the unavailability of a temperature recording chart was observed. This calls for continuous supportive supervision of immunization nurse, provision of functional fridge-tags for continuous monitoring of vaccine storage temperatures as proposed by Falcon et al. [29] and provision of temperature monitoring charts to plot the observed values.

In contrast, the percentage of functional refrigerators in this study was higher than the one found in Ethiopia (76.7%) by Bogale et al. [25]. This difference could be due to the socioeconomic difference between Kenya and Ethiopia. However, 12.29% of facilities had no refrigerator, which increases missed opportunities in immunization.

Adherence to a routine planned preventive maintenance of refrigerators in Kenya was comparably as low as in Ethiopia [31]. This could be due to reduced funding and non-inclusion of preventive maintenance in the hospital annual workplans. Besides, shortage of biomedical staff in the two LMICs could also be a contributing factor.

Stock-outs were a similar observation in both this study and that of Lucy et al. conducted in Nairobi city [32]. In fact, facilities in urban and rural areas rely on the same vaccine supply chain system from the central stores.

Limitations of this study included the low coverage of the study population and the non-representative geographical distribution of facilities; therefore, these results should not be generalized at the entire rural areas of Kenya. Devolution has resulted in urbanization whereby rural areas are upgrading in terms of accessibility due to infrastructural improvements. In addition, missing data and social desirability bias may have occurred during the administration of the structured questionnaires.

## Conclusion

The findings of this study have revealed that there is suboptimal vaccine storage and distribution practices in the last mile, to rural health facilities. These gaps in immunization supply chain management in Turkana county may contribute to vaccine stock outs hindering optimal immunization service delivery in health facilities of the county.

## Data Availability

The datasets used and/or analyzed in this study are available from the corresponding author on reasonable request.

## Abbreviations

COVAX: COVID-19 vaccines global access
EPI: Expanded program on immunization
ERC: Ethics Review Committee
FEFO: First expiry-first-out
KNH: Kenyatta National Hospital
LICs: Lower income countries
LMICs: Lower middle-income countries
MVDP: Multi-vial dosing policy
NACOSTI: National Commission for Science Technology and Innovation
UNICEF: United Nations Children’s Fund
UON: University of Nairobi
WHO: World Health Organization
VVM: Vaccine vial monitor
